# Comparing kinematic asymmetry and lateral step-down test scores in healthy, chronic ankle instability, and patellofemoral pain syndrome female basketball players: a cross-sectional study

**DOI:** 10.1038/s41598-023-39625-1

**Published:** 2023-07-31

**Authors:** Mahsa Emamvirdi, Mahdi Hosseinzadeh, Amir Letafatkar, Abbey C. Thomas, Thomas Dos’Santos, Nicola Smania, Giacomo Rossettini

**Affiliations:** 1 Department of Biomechanics and Sport Injuries, Kharazmi University, Tehran, Iran; 2 Department of Sport Injuries and Corrective Exercises, Sport Sciences Research Institute, No. 3, 5th Alley, Miremad Street, Motahhari Street, P O Box: 1587958711, Tehran, Iran; 3 Sports Injury and Corrective Exercises, Faculty of Physical Education and Sport Sciences, Kharazmi University, Tehran, Iran; 4 Department of Applied Physiology, Health, and Clinical Sciences, University of North Carolina at Charlotte, Charlotte, NC USA; 5 Department of Sport and Exercise Sciences, Musculoskeletal Science and Sports Medicine Research Centre, Manchester Metropolitan University, Manchester, UK; 6 Manchester Institute of Sport, Manchester Metropolitan University, Manchester, UK; 7 Department of Neurosciences, Biomedicine and Movement Sciences, University of Verona, Verona, Italy; 8 Neuromotor and Cognitive Rehabilitation Research Centre (CRRNC), University of Verona, Verona, Italy; 9 School of Physiotherapy, University of Verona, Verona, Italy

**Keywords:** Risk factors, Predictive markers, Prognostic markers, Population screening, Chronic pain

## Abstract

We aimed to understand whether ankle dorsiflexion range of motion (ROM) and dynamic knee valgus (DKV) kinematic inter-limb asymmetries would be associated with the Lateral Step-Down Test (LSD) in basketball players with chronic ankle instability (CAI), patellofemoral pain (PFP) and healthy controls (HC). An observational cross-sectional study with a between-subject design was employed. Female basketball athletes with CAI (n = 20), PFP (n = 20) and HC (n = 20) were recruited. Ankle dorsiflexion-ROM, DKV angle during a single-limb squat, and LSD quality were measured bilaterally. The Asymmetry index (ASI) was calculated to identify between-limb percentage imbalances. The correlation matrix between the tasks was calculated. Ankle dorsiflexion-ROM was less in the CAI and PFP than in the HC group regardless of limb (p < 0.001). DKV angle was greater in the CAI and PFP than in the HC group bilaterally (p < 0.001). LSDs were similar between the PFP and CAI groups (p = 0.698) but worse than the HC group (p = 0.001). The ASI showed asymmetry across all tasks (p < 0.001), with the greatest asymmetry for the DKV angle. The correlation matrix between tasks on both limbs was significant (p < 0.05). Our findings suggest significant asymmetries in ankle dorsiflexion-ROM and frontal plane knee control are present in female basketball athletes with CAI and PFP, and thus, highlights need to evaluate and reduce limb asymmetries in these populations.

## Introduction

Many health benefits accrue from consistent physical activity across the lifespan. Participation in physical activities, however, carries an inherent risk for acute and chronic musculoskeletal injury^1^. Musculoskeletal injuries, particularly of the lower extremity, cause short-term disability, interfere with participation in physical activity, and are associated with joint disorders in later life^2^. Thus, the general goal of preventing acute and chronic musculoskeletal injuries and reducing the associated burden among high-risk individuals is of considerable interest. Many studies report a higher prevalence of musculoskeletal pain among females in the general population^3^. When scholastic and colleagiate athletes are considered, females continue to experience greater rates of injury, particularly to the knee and ankle experiencing higher prevalences of Patellofemoral pain (PFP) and chronic ankle instability (CAI) in weight-bearing sports (e.g. basketball) compared to their male counterparts^4^. PFP is a multifactorial clinical condition resulting from abnormal patellofemoral joint loading, increasing joint stress and producing retropatellar pain^5^. Conversely, CAI represents both mechanical and functional instability of the ankle joint resulting in repetitive bouts of instability following an initial lateral ankle sprain injury^6^. PFP and CAI represent two of the most prevalent musculoskeletal injuries among physically active individuals^4^ and, while these conditions affect different joints, a commonality among them is that patients with these musculoskeletal disorders experience aberrant movement patterns that may contribute to/arise from the disorder’s presence^7^.

Previous studies have reported that dynamic knee valgus (DKV), an aberrant movement pattern during weight-bearing activities, is a significant risk factor in patients with PFP^7^. Though few researchers have investigated DKV angle in persons with CAI, those who have studied it found DKV angle to be greater in this population compared to their health counterparts^8,9^. Accordingly, an increased DKV angle is not only associated with painful knee conditions but can also be related to dysfunctions in other lower limb joints (e.g. the ankle)^10^. Reduced ankle dorsiflexion range of motion (ROM) has been reported as a risk factor for some conditions, such as patellar tendinopathy^11^, Achilles tendinopathy^12,13^, chronic ankle instability^14^, metatarsal stress fractures^11^, and anterior knee pain^15^.

In a closed kinetic chain, the ankle acts as a firm base of support such that its movement restriction and/or instability can affect the function of proximal joints^14^. Therefore, restriction of ankle dorsiflexion ROM represents a possible risk factor for excessive DKV angle^16,17^ during squat and jump landing tasks^18^ and is linked to injurious landing mechanics^19^. With restricted ankle dorsiflexion ROM, individuals may try to compensate with movement in the frontal or transverse plane throughout the overall kinetic chain^16,17^, thus, creating an increased DKV angle^16,17,20^. This compensation may present as foot pronation^20^, internal tibial rotation^20^, hip internal rotation and adduction^21^, pelvic drop^15^, or gastrocnemius and soleus tightness^22^. Patients with PFP^22^ and CAI^14^ commonly display a limited ankle dorsiflexion ROM with adverse functional consequences for the knee joint biomechanics during landing^9^, ascending/descending stairs, squatting, jumping and running^23^. Restricted dorsiflexion will also increase impact forces / mechanical loads and redistribute them proximally to the knee and hip in weight-bearing activities such as the Lateral Step-Down test (LSD)^14^. The LSD is a simple, clinician-friendly tool designed to assess lower extremity movement quality during a functional activity^24^. The test allows clinicians to identify faulty movement patterns and evaluate the trunk, hip and knee behaviour during the task^24^. Since its creation, the LSD has been used to assess patients’ quality of movement with CAI and PFP^14,15^. There are several advantages of using LSD to assess movement quality, mainly because it is a quick and easy test to perform in a clinical environment^24^. Moreover, the performance of the LSD score is influenced by a reduced ankle dorsiflexion ROM in individuals with PFP and CAI^14,15^. Therefore, ankle dorsiflexion ROM represents a key assessment when patients demonstrate a lower movement quality during an LSD^14,15^.

Quantifying neuromuscular control between legs (e.g. reduced function or performance in one limb compared to the other) is critical to identify individuals potentially at risk of injury, establishing when an athlete can return to play following injury, and optimizing strength and conditioning training^25^. Neuromuscular asymmetry of the lower limbs is associated with potential injury and can be used to predict future injury or re-injury^26^. Inter-limb asymmetries (ILA) may potentially place both legs at an increased risk of injury in sports; the strong leg may sustain excessive stress due to high dependence and loading (i.e. overuse), whereas the weak leg may be compromised to a potentially lower load tolerance capacity^27^. In addition, ILA have been associated with an increased risk of sports injury because the asymmetries may result in unequal force attenuation or a loss of frontal plane stability, which are important to sustain the impacting forces^26^. For example, research has examined lower ILA, mainly in healthy basketball players, during functional movements (e.g. cutting, pivoting, running)^28^, leaving uncertainty about their role in athletes with CAI and PFP. Individuals with PFP may have gluteus medius muscle activation asymmetry, which may be associated with pain severity^29^. Also, Nakagawa et al.^30^ showed that male military recruits with greater asymmetry on the Y-Balance Test (YBT) posterolateral direction and Frontal Plane Knee Projection Angle (FPKPA) during single-leg squat were at a greater risk of developing PFP^30^. However, Plastaras et al.^31^ showed early stages of unilateral PFP in female runners was not associated with hip abduction strength asymmetry. Regarding patients with CAI, the asymmetry between right and left anterior reach distances (> 4 cm) and limitations in posterolateral reach distances (< 80% normalized reach distance) represent risk factors for lateral ankle sprains and may contribute to CAI^32^. Tajdini et al. showed that patients with CAI walk with greater ILA in vertical ground reaction force and muscle activity across the gait cycle compared to the non-CAI group^33^.

Although limited dorsiflexion ROM and its effect on excessive DKV and lower movement quality have been reported in PFP and CAI^10,14^, to our best knowledge, there is a lack of research concomitantly investigating all three tasks (DKV angle, ankle dorsiflexion ROM and movement quality) in both lower limbs (injured and contralateral) among basketball players presenting PFP and CAI. From a clinical perspective, a better understanding of these phenomena would inform researchers and clinicians (e.g. physical therapists and physicians) to decide on the assessment, treatment evolution, and improving movement control of these patients considering both limbs and functional relationship of the lower kinetic chain^34^.

Therefore, this study aims to understand whether dorsiflexion ROM and DKV kinematic ILA would be associated with the LSD scores in basketball players with CAI or PFP compared to healthy controls, and whether differences exist in these measures between populations. We hypothesized that: (1) there would be significant differences in DKV angle, ankle dorsiflexion ROM, and LSD scores in CAI and PFP compared to healthy controls; (2) there would be no significant difference between CAI and PFP in DKV angle, ankle dorsiflexion ROM, and LSD scores; (3) ankle dorsiflexion ROM and DKV asymmetry would be associated with LSD scores asymmetry in PFP and CAI; and (4) there would be an inverse association between ankle dorsiflexion ROM and LSD scores and a direct relation between DKV angle and LSD scores.

## Materials and methods

### Study design

This observational cross-sectional study was conducted and reported following the Strengthening the Reporting of Observational Studies in Epidemiology (STROBE) guidelines. The study conformed to the Declaration of Helsinki. All participants provided written informed consent. The ethics board of the Sport Science Research Institute (IR.SSRC.REC.1400.120) approved this study.

### Participants

Participants in this study were recreational, female, basketball athletes recruited from sports clubs in Iran. Participants were between 20 and 30 years of age (CAI: 22.70 ± 1.94 years, PFP: 23.05 ± 2.08 years, Healthy: 22.75 ± 2.31 years). The population of female basketball players were chosen due to the high prevalence of CAI and PFP^4,35^. A physiotherapist with 13 years of experience in diagnosing and treating patients with musculoskeletal disorders assessed all the athletes, classifying them eligible when presenting with unilateral CAI, unilateral PFP or healthy conditions based on the inclusion and exclusion criteria reported in Table 1. Further, the physiotherapist confirmed the absence of PFP in the CAI group and the absence of CAI in the PFP group. Patients of the CAI and PFP groups were matched in age with healthy individuals. Moreover, during the research period, the participants were not receiving medical or rehabilitative care for CAI and PFP.

**Table 1:** Inclusion and exclusion criteria.

	Inclusion criteria	Exclusion criteria
CAI group	- CAI was defined as incurring at least one ankle sprain and at least one subsequent episode of giving way occurring at least 12 months before the study -We used the Iranian version of CAIT, FAAM– ADL and the FAAM–Sport -Reporting a score: < 24 on the (CAIT)^36^, < 90% on the FAAM– ADL < 80% on the FAAM–Sport^36,37^	- A recent history (< 6 months) of lower extremity injury/surgery (including lateral ankle sprain), diagnosis of ankle osteoarthritis, history of ankle surgery involving intra-articular fixation, or current pregnancy^14^
PFP group	-Participants were included in the PFP who showed anterior knee pain for at least three months during the performance of at least two of the following tasks: ascending and descending stairs, squatting, running, jumping or remaining seated for a long time, besides showing a minimum score of three points in the NPRS^38^ -Participants also must have presented with an insidious onset of symptoms unrelated to trauma and a positive Clark test^39^	-Tenderness to palpation of the patellar tendon, the inferior pole of the patella, or tibial tubercle as the primary complaint. Tenderness to palpation of the patellar tendon, the inferior pole of the patella, or tibial tubercle as the primary complaint^10^ -Other diagnoses of the knee including: patellar tendinitis, iliotibial band syndrome, Osgood- Schlatter’s disease, Sinding-Larsen’s Johansson’s disease, fracture, or ligamentous injuries. Prior knee surgery, history of patellar subluxation or dislocation^10^
Control healthy group	-Participants had to be free of lower extremity symptoms -Without any history of ankle sprains -No pain in the NPRS -Negative Clark test	-History of pathology involving the comparable knee, ankle or other joints of the lower extremity -Reported any history of lower extremity surgery, neuropathies, diabetes, balance disorder, Raynaud’s diseases, cold-induced circulatory problems, and other conditions known to affect balance

*CAI* chronic ankle instability, *PFP* patellofemoral syndrome, *CAIT* FAAM-*ADL* foot and ankle ability measure-activities of daily, *CAIT* cumberland ankle instability tool, *FAAM*-*ADL* foot and ankle ability measure-activities of daily, *FAAM*-*sport* foot and ankle measure-sport, *NPRS* numeric pain rating scale.

### Sample size

G-Power software (G*Power©, University of Dusseldorf, Germany) was used to estimate the sample size according to a recent similar study that assessed DKV angle and LSD. The analysis revealed that to perform repeated measures, between factors Multivariate Analysis of Variance (MANOVA) with an effect size of 0.30, power of 0.30, and alpha of < 0.05, we would need at least 19 participants. To account for potential dropout, we determined a minimum sample size of 60 participants was required to be assigned into three groups: 20 participants with CAI, 20 with PFP, and 20 healthy females.

### Setting and procedure

In the pre-screening phase, the research group distributed recruitment flyers by hand (brochures) to basketball clubs’ meetings in the metropolitan area of Tehran. Before participating in the study, all athletes were briefed about the objectives and read and signed the informed consent form. Then, the included participants attended a baseline assessment as follows:

1. The CAI group completed a series of patient-reported outcomes surveys to confirm a history of significant ankle sprains/giving way/recurrent ankle sprains, including the Iranian version of the Cumberland Ankle Instability Tool Questionnaire (CAIT)^36^ and the Persian version of Foot and Ankle Ability Measure (FAAM) for activities of daily living (ADL) and SPORTS subscales^37^.
2. The PFP group were assessed by the Numerical Pain Rating Scale (NPRS)^38^ and the Clark test^39^.
3. The Healthy group completed the CAIT^36^, FAAM-ADL and FAAM-SPORTS^37^, NPRS^38^ and Clark test^39^.

Next, participants reported their age and involved limb, had their height (cm) and body mass (kg) measured using a tape measure and a digital scale, respectively, and their body mass index (BMI) calculated.

Moreover, the ankle dorsiflexion ROM and DKV angle were measured, and the LSD was performed for both lower limbs. The first limb measured (right or left) was randomized, but ankle dorsiflexion ROM measures were always obtained before the DKV angle and LSD assessments. All the assessments were conducted with the participants barefoot at Kharazmi University in January 2020. An examiner with 13 years of experience that received 5 h of training in ankle dorsiflexion ROM, DKV angle, and LSD assessments performed all the measurements. The examiner was blinded to the group allocation to avoid bias during the evaluation.

### Instruments and measurements

#### Ankle dorsiflexion ROM

**Figure 1:**
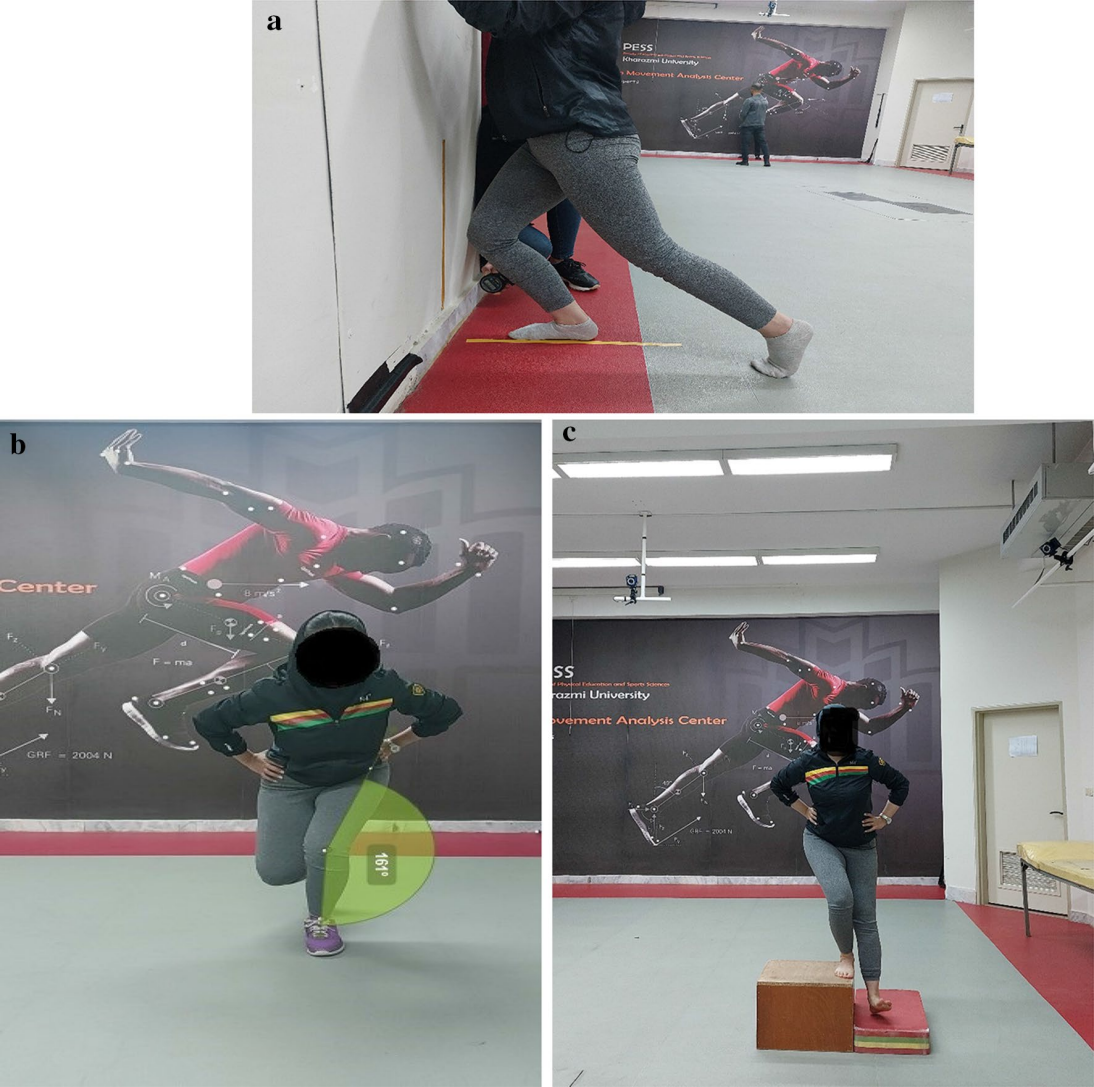
(**a**) Weight-bearing lunge test ankle dorsiflexion ROM. (**b**) Dynamic Knee Valgus (DKV) angle measurement. (**c**) Lateral step-down test (LSD).

A digital inclinometer was used to record weight-bearing ankle dorsiflexion ROM by measuring the tibia angle to the ground during a two-point staggered upright position^40^. Ankle dorsiflexion ROM in the weight-bearing lunge (WBLT) was measured with an Acumar™ digital inclinometer (model ACU360 Lafayette Instrument Company, Lafayette, IN, USA Single Digital Inclinometer). The participant put the tested foot along a 50-cm-long line drawn on the ground, and a continuous 60-cm-long line was drawn on a wall. A test taker placed an inclinometer on the anterior aspect of the tibia, 15 cm distal to the tibial tuberosity, so that the line bisected the heel, and the second toe was on the line. The subject lunged forward and directed their patella as close as the line drawn on the wall while the heel remained in contact with the floor. This puts the ankle joint in maximal dorsiflexion. Once maximal dorsiflexion was reached, the ankle dorsiflexion ROM was recorded (Fig. 1a)^40^. Three assessments were recorded, and the average of the three trials was used for further analysis^41^. This method results in higher reliability coefficients (ICC = 0.96 to 0.99), representing a valuable assessment strategy^41^. In this study, the tests were conducted for the intraclass reliability of the examiner, resulting in a correlation coefficient (within-session reliability) of 0.95 (0.92–0.97).

#### Dynamic knee valgus (DKV) angle measurement

We measured the DKV angle during a single-leg squat. The single-leg squat was chosen because it allows for easy visualization of poor neuromuscular control (e.g. increased DKV angle) in patients with PFP and CAI^7,8^. Participants were asked to stand on the test limb, facing the video camera and then to squat down at an angle of at least 45° knee flexion but not greater than 60°, for 5 s. The knee flexion angle was checked during practice trials (maximum of three) using a standard goniometer observed by the same examiner throughout all trials^42^. Trials were only accepted if the participant squatted within the desired range of knee flexion and maintained their balance while keeping their hands on their iliac crests throughout the trial^42^. Two-dimensional videos of the single-leg squat were captured using a Canon Vixia HF R42 digital camera (Canon USA), sampling at 60 frames per second. The camera was placed at the height of the subject’s knee, 3 m anterior to the participant’s squatting area, and aligned perpendicular to the frontal plane. DKV angle was calculated as the angle formed between the thigh and shank segments (Fig. 1b). Specifically, a line drawn from the anterior superior iliac spine to the midpoint of the knee, bisecting the thigh, defined the thigh segment. A line drawn from the knee's midpoint to the ankle’s midpoint defined the shank segment. The DKV angle was calculated as 180° minus the angle between the thigh and shank segments^42^. DKV angles were obtained at peak knee flexion for each trial, determined visually by the investigator. The same individual who experienced palpation placed the markers on all participants. The average DKV angle value for both measures from three trials was analyzed^42^ and processed using Kinovea Software (v0.8.15; Kinovea Open Source Project, www.kinovea.org). Within-day ICCs showed good reliability and ranged from 0.59 to 0.88, and between-days ICCs were good to excellent, ranging from 0.72 to 0.91^42^. In this study, the tests were conducted for the intraclass reliability of the examiner, resulting in a correlation coefficient (within-session reliability) of approximately DKV 0.92 (0.90–0.95).

#### Lateral step-down (LSD)

The LSD measured the quality of functional movements^24^. Participants stood in a unilateral stance atop a 20-cm step with the medial border of their foot near the stair edge and their hands on the hips. The limb not being tested was held off the step over the floor. In the next stage, they were asked to slowly descend the stair by flexing the test limb knee so that the contralateral heel touched the ground and returned to the initial position. This maneuver was repeated for 5 repetitions. Participants received no feedback on performing the task and no information on performance errors. A video camera (Canon Vixia HF R42 digital camera [Canon USA]; 60 fps sampling rate) aligned with the frontal plane was used to record the assessments for subsequent scoring (Fig. 1c). There were no limits on the number of times a video was watched or constraints on the playback speed when scoring the LSD. All participants began with a score of 0, and 1 point was added for each error committed^15^. Errors included: picking up hands from the hips, rotating or lifting the pelvis, flexing the trunk, moving into DKV (tibia protrusion deviating outwards from the second finger), and inability to sustain unilateral balance (e.g. bearing weight given the opposite limb)^15^. If the DKV angle exceeded the foot midline, an extra point was given for a total of 6 points. Higher scores (more errors) indicated poorer movement quality (0–1: “good”, 2–3: “moderate”, and 4–6: “poor”)^15^. Pilot data from our lab suggest inter-rater agreement on the LSD to be substantial (kappa = 0.74), which aligns with previously established values in the literature (k = 0.67- 0.81)^43^.

### Data analysis

The Asymmetry index (ASI) was calculated to identify functional imbalances between limbs using the following formula:^44^ ASI = (contralateral (or left) leg- involved (or right) leg/contralateral leg) × 100.

To confirm that inter-limb differences are meaningful, we calculated the coefficient of variation (CV)^45^ as (SD/mean) × 100 for each participant and then averaged across all participants.

We considered the right side as the involved leg for the healthy group when calculating the ASI.

### Statistical analysis

The independent variables in this study were group (PFP, CAI, or healthy) and limb (involved, contralateral). Dependent variables were LSD, DKV angle, and ankle dorsiflexion ROM. The SPSS software (ver. 23 for Windows; SPSS Inc., Chicago, IL, USA) was used to analyze the data collected in this study. Descriptive statistics were adopted to summarize the data with measures of central tendency. The Shapiro–Wilk and also Kolmogorov–Smirnov tests assessed the normal distribution of the tasks. A Two-way mixed model ANOVA was used to compare the interaction of group on limb on each dependent variable. The Scheffe test was used for multiple comparisons for the post hoc test. Paired *t* tests were adopted to further elucidate differences between the involved and contralateral legs in three tasks (ankle dorsiflexion ROM, DKV angle, and LSD). For non-parametric data (LSD), the Kruskal Wallis and Mann–Whitney *U* tests were used to determine the difference between groups. To determine the association between tasks on the involved and contralateral limbs, the relationship between ankle dorsiflexion ROM measures, DKV angle, and the LSD score was determined using a Spearman rank correlation coefficient. Correlation values were interpreted as follows:  < 0.25 little or no relationship, 0.25–0.50 fair relationship, 0.50–0.75 moderate to good relationship,  > 0.75 good to excellent relationship^46^. The alpha value was set at p < 0.05 for all analyses. Finally, effect sizes (ES) (bias-corrected Hedges’ g) and 95% confidence interval (CI) were also computed to estimate the precision and magnitude of group differences, given the multiple *t* tests ran. Effect sizes were interpreted as trivial (≤ 0.19), small (0.20–0.59), moderate (0.60–1.19), large (1.20–1.99), very large (2.00–3.99), and extremely large (≥ 4.00) for paired and independent comparisons^47^.

### Ethics declarations

The study was approved by Research Ethics Committees of Iran institute of sport science (Approval IR.SSRC.REC.1400.120). This study was performed in accordance with the standards of ethics outlined in the Declaration of Helsinki.

## Results

### Participants

**Figure 2:**
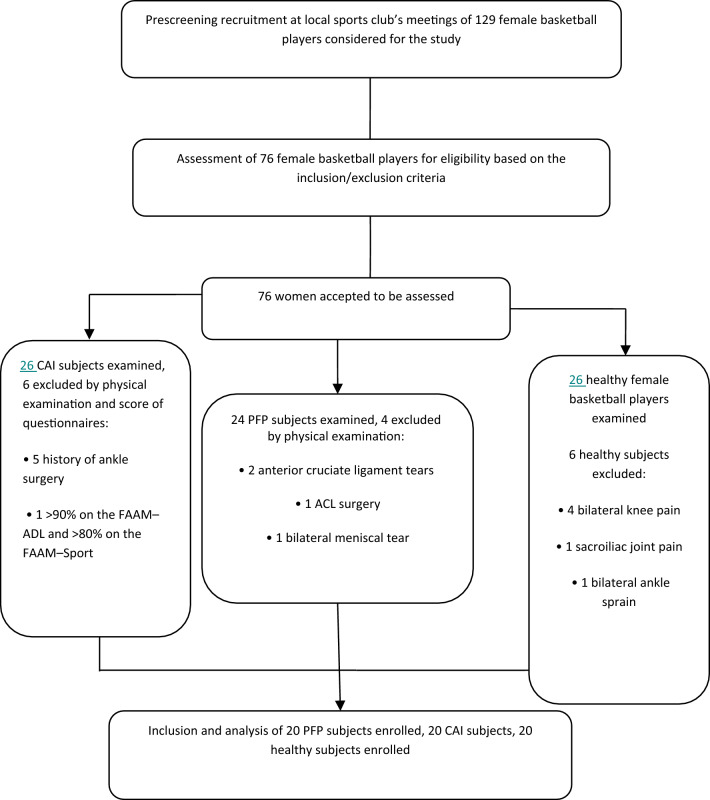
Flowchart for enrollment of the participants in the current study.

One hundred and twenty-nine basketball players were screened, 76 participants were assessed for eligibility based on the inclusion/exclusion criteria. Next, we selected 24 participants from the PFP group, 26 from the CAI group, and 26 from the healthy group. Finally, considering the dropout and based on the exclusion criteria, we included 60 participants (20 participants in each group). The flowchart of the participants’ selection is reported in Fig. 2.

### Descriptive data

There were no significant differences between groups in age, height, mass, and BMI (p > 0.05; Table 2). The CAI group reported lower CAIT, FAAM-ADL, and FAAM-Sport scores (p < 0.001) compared to the healthy group (Table 2). The PFP group reported higher NPRS (p < 0.001) than the healthy group.

**Table 2:** Participant characteristics at the baseline (mean ± SD).

Groups	CAI (n = 20)	PFP (n = 20)	Healthy (n = 20)	Kolmogorov–Smirnov test	Shapiro–Wilk’s test	One-way Anova (p-value)	Multiple difference comparison					
							CAI vs PFP	ES	CAI vs Healthy	ES	PFP vs healthy	ES
Age (y)	22.70 ± 1.94	23.05 ± 2.08	22.75 ± 2.31	0.109	0.056	0.853	0.932	0.174	0.357	0.023	0.445	0.136
Mass (kg)	56.65 ± 3.49	56.25 ± 2.80	57.60 ± 3.13	0.097	0.056	0.388	0.128	0.126	0.517	0.286	0.318	0.454
Height (cm)	166.65 ± 4.45	166.90 ± 3.98	168.85 ± 3.89	0.200	0.196	0.190	0.513	0.059	0.403	0.526	0.842	0.724
BMI (Kg/m^2^)	20.40 ± 1.22	20.19 ± 0.68	20.19 ± 0.64	0.200	0.560	0.679	0.60	0.212	0.041	0.215	0.817	0.000
Involved limb (right/left)	14/6	15/5	–	–	–	–	–	–	–	–	–	–
CAIT	17.30 ± 2.38	–	30.00 ± 0.00	–	–	0.000	–	–	0.000	–	–	–
FAAM-ADL	76.36 ± 2.89	–	100.00 ± 0.00	–	–	0.000	–	–	0.000	–	–	–
FAAM-Sport	69.01 ± 4.96	–	100.00 ± 0.00	–	–	0.000	–	–	0.000	–	–	–
NPRS	–	5.95 ± 1.05	0.00 ± 0.00	–	–	0.000	–	–	–	–	0.000	–

*CAI* chronic ankle instability, *PFP* patellofemoral syndrome, *n* number, *y* year, *kg* kilogram, *cm* centimeter, *BMI* body mass index, *CAIT* cumberland ankle instability Tool, *FAAM*-*ADL* foot and ankle ability measure-activities of daily, *FAAM* sport foot and ankle measure-sport, *NPRS* numeric pain rating scale, *ES* effect size.

### Outcome data

The results demonstrated differences between the three groups for ankle dorsiflexion ROM, DKV angle and LSD in both the involved and contralateral lower limbs (p < 0.001; Tables 3 and 5). In addition, Two -way mixed model ANOVA revealed significant differences in ankle dorsiflexion involved*LSD contralateral in PFP, LSD contralateral* ankle dorsiflexion ROM contralateral in group CAI, and LSD involved* DKV angle involved in CAI group. Regarding the other factors in the three groups, there was no significant difference (Table 4).

**Table 3:** Result of one-way ANOVA on data in healthy participants and those presenting with CAI and PFP.

Variable	Kolmogorov–Smirnov test	Shapiro–Wilk’s test	Group	Kolmogorov–Smirnov test	Shapiro–Wilk’s test	mean ± SD	ANOVA	Multiple comparisons differences (p)					
								CAI vs PFP	ES	CAI vs healthy	ES	PFP vs healthy	ES
Ankle dorsiflexion ROM involved	0.200	0.167	CAI	0.200	0.858	37.99 ± 3.36	F = 17.84 p-value = 0.001	− 2.98 (0.153)	− 0.59	− 8.86 (0.001)	− 2.29	− 5.88 (0.001)	− 1.10
			PFP	0.200	0.427	40.97 ± 6.22							
			Healthy	0.200	0.927	46.90 ± 4.33							
Ankle dorsiflexion ROM contralateral	0.200	0.306	CAI	0.116	0.054	44.44 ± 3.62	F = 12.90 p-value = 0.001	4.400 (0.011)	0.97	− 2.67 (0.174)	− 0.67	− 7.07 (0.001)	− 1.47
			PFP	0.200	0.466	40.04 ± 5.29							
			Healthy	0.200	0.313	47.11 ± 4.25							
DKV involved	0.063	0.035	CAI	0.200	0.887	16.36 ± 2.62	F = 82.03 p-value = 0.001	− 0.50 (0.583)	− 0.16	7.70 (0.001)	3.43	8.20 (0.001)	3.12
			PFP	0.200	0.295	16.86 ± 3.26							
			Healthy	0.200	0.358	8.66 ± 1.78							
DKV contralateral	0.200	0.236	CAI	0.200	0.305	13.24 ± 1.85	F = 53.90 p-value = 0.001	− 1 (0.647)	− 0.40	5.16 (0.001)	2.81	6.16 (0.001)	2.46
			PFP	0.73	0.104	14.26 ± 3.04							
			Healthy	0.197	0.175	8.09 ± 1.81							
LSD involved	0.000	0.001	CAI	0.000	0.006	3.45 ± 0.94	F = 70.28 p-value = 0.001	0.05 (0.698)	0.04	2.90 (0.001)	3.83	2.85 (0.001)	3.34
			PFP	0.000	0.002	3.40 ± 1.09							
			Healthy	0.000	0.000	0.55 ± 0.51							
LSD contralateral	0.000	0.000	CAI	0.000	0.000	1.40 ± 0.59	F = 27.36 p-value = 0.001	− 0.7 (0.049)	− 0.76	1.15 (0.001)	2.20	1.85 (0.001)	2.10
			PFP	0.000	0.002	2.10 ± 1.16							
			Healthy	0.000	0.000	0.25 ± 0.44							

*CAI* chronic ankle instability, *PFP* patellofemoral syndrome, *ROM* range of motion, *DKV* dynamic knee valgus, *LSD* lateral step-down test, *ES* effect size, *SD* standard deviation, *ROM* range of motion.

**Table 4:** Results of the two-way mixed model ANOVA.

Dependent variables	LSD involved			LSD contralateral			LSD involved*LSD contralateral		
	CAI	PFP	Healthy	CAI	PFP	Healthy	CAI	PFP	Healthy
Ankle dorsiflexion ROM involved	0.253	0.146	0.570	0.306	0.042*	0.124	0.732	0.837	–
Ankle dorsiflexion ROM contralateral	0.264	0.730	0.739	0.016*	0.404	0.269	0.841	0.823	–
DKV involved	0.016*	0.669	0.093	0.305	0.120	0.684	0.888	0.848	–
DKV contralateral	0.051	0.669	0.144	0.751	0.120	0.614	0.934	0.753	–

Data are presented as P-values.

The mean difference is significant at the 0.05 level, *CAI* chronic ankle instability, *PFP* patellofemoral syndrome, *ROM* range of motion, *DKV* dynamic knee valgus, *LSD* lateral step-down test, *ROM* range of motion.

* indicates significant difference at the p <  0.05 level.

### Ankle dorsiflexion ROM

**Figure 3:**
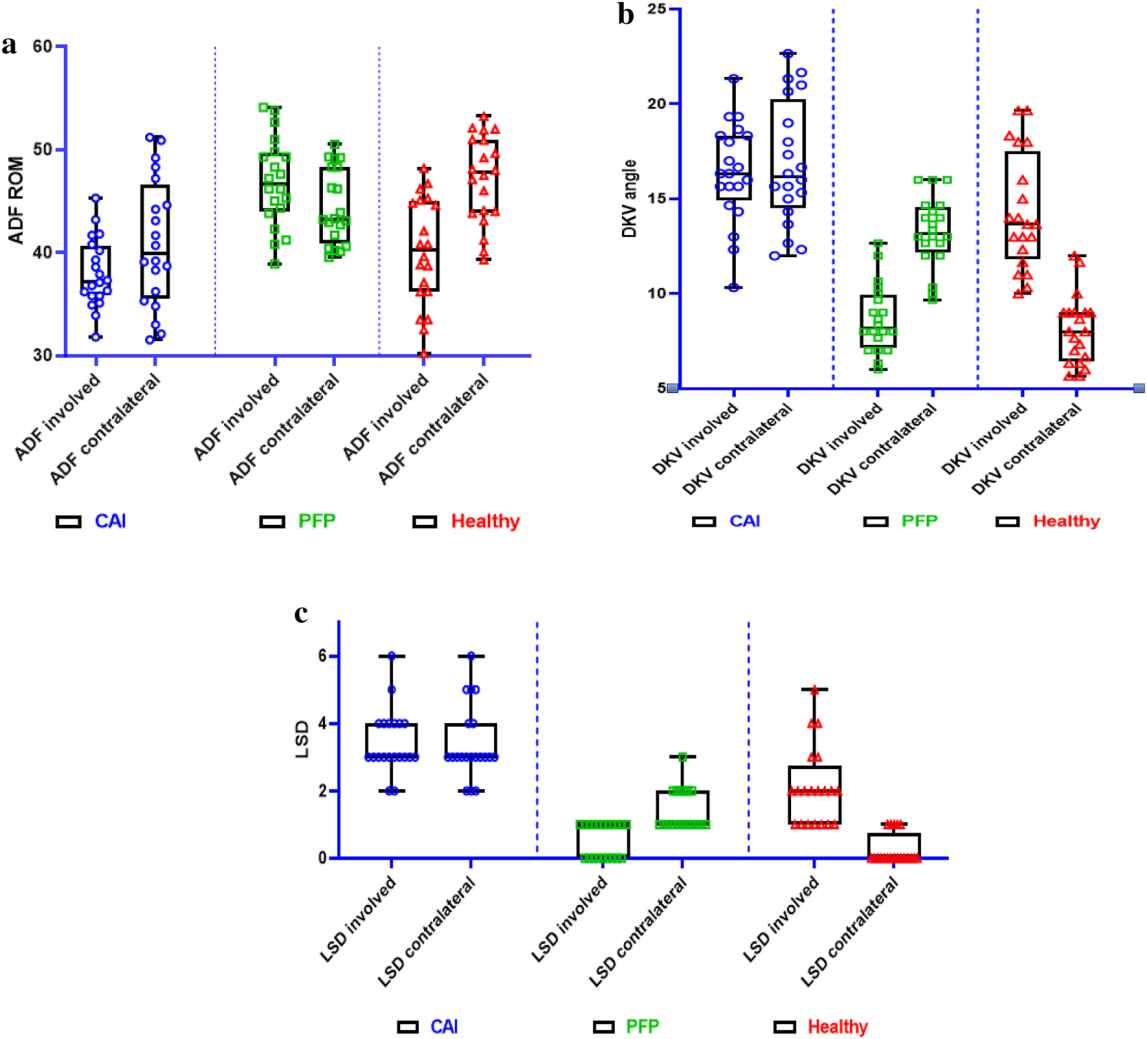
(**a**) Comparison of the ankle dorsiflexion ROM in groups with CAI, PFP and the healthy group. *ADF ROM* ankle dorsiflexion range of motion, *CAI* chronic ankle instability, *PFP* patellofemoral pain. (**b**) Comparison the DKV angle in the groups with the CAI, PFP, and the healthy group. *DKV* dynamic knee valgus angle, *CAI* chronic ankle instability, *PFP* patellofemoral syndrome. (**c**) Comparison of the LSD scores in the groups with the CAI, PFP and the healthy group. *LSD* lateral step-down test, *CAI* chronic ankle instability, *PFP* patellofemoral syndrome.

Ankle dorsiflexion ROM was significantly greater in healthy controls compared to CAI and PFP groups with very large and moderate effect sizes, respectively (Fig. 1a, Tables 3 and 4). The contralateral ankle dorsiflexion ROM was significantly greater in the CAI compared to the PFP group with a moderate effect size and significantly greater in healthy controls compared to the PFP group with a large effect size (Fig. 3a, Tables 3 and 4).

### DKV

Contralateral limb DKV angle was significantly greater in the CAI and PFP groups compared to healthy controls with very large effect sizes. (Fig. 3b, Tables 3 and 4).

### LSD

A significant main effect of DKV involved was observed for LSD involved in individuals with CAI (Table 4). Involved limb DKV angle was significantly greater in the CAI and PFP groups than in healthy controls with large effect sizes (Tables 3 and 4). A significant main effect of ankle dorsiflexion ROM was observed for LSD contralateral in individuals with PFP. A significant main effect of ankle dorsiflexion ROM contralateral was also observed for LSD contralateral in individuals with CAI (Table 4). LSD scores revealed no significant difference in movement quality based on the test in the involved limb in the CAI group compared to the PFP group with a trivial effect size. However, three scores differed between groups with a very large effect size (Tables 3 and 4). Contralateral limb LSD scores were 1.15 points greater in the CAI compared to the healthy groups with a very large effect size. Similarly, contralateral LSD scores were 1.85 points greater in the PFP group, compared to the healthy group with a very large effect size (Fig. 3c, Tables 3 and 4).

### Asymmetry index

Testing of homogeneity of variance revealed that data were not normally distributed. There was a significant difference in asymmetry index across all tasks (ankle dorsiflexion ROM, DKV angle) in each group separately (Table 5). The greatest asymmetry was presented in the DKV angle for the CAI and PFP groups (− 23%, − 18%, respectively). It is worth noting that PFP and healthy groups in ankle dorsiflexion ROM had a CV value greater than the asymmetry score; thus, as a group, these athletes did not have a meaningful between-limb imbalance in ankle dorsiflexion ROM.

**Table 5:** Asymmetry between limbs.

ASI (%)	Group			Total	P-value	Multiple comparison (post-hoc test) on asymmetry between limbs			
	CAI	PFP	Healthy			(I) Group	(J) Group	I-J mean ± SD ASI (%)	I-J p-value
Ankle dorsiflexion ROM mean ± SD	14.39 ± 5.57*^†^	− 2.46 ± 9.09	− 0.43 ± 2.27	4.12 ± 9.0.66	0.001, F = 41.02	CAI	PFP	16.86 ± 2.38	< 0.001*
							Healthy	13.96 ± 1.34	< 0.001*
ES	1.94	0.16	0.03	3.16		PFP	CAI	− 16.86 ± 2.38	< 0.001*
							Healthy	− 2.89 ± 2.09	0.440
CV%	11.09 ± 4.56	5.82 ± 2.89	1.38 ± 0.76	6.10 ± 5.06		Healthy	CAI	− 13.96 ± 1.384	< 0.001*
							PFP	2.89 ± 2.09	0.440
DKV angle (°) mean ± SD	− 23.36 ± 8.43^†^	− 18.72 ± 4.95†	− 7.86 ± 10.05	− 16.65 ± 10.30	0.001, F = 19.27	CAI	PFP	− 4.63 ± 2.18	0.119
							Healthy	− 15.49 ± 2.93	< 0.001*
ES	1.37	0.82	0.31	2.32		PFP	CAI	4.63 ± 2.18	0.119
							Healthy	− 10.85 ± 2.50	< 0.001*
CV%	14. 62 ± 4.80	12.04 ± 2.95	7.02 ± 4.44	11.22 ± 5.17		Healthy	CAI	15.49 ± 2.93	< 0.001*
							PFP	10.85 ± 2.50	< 0.001*

The mean diffrence is siggnificant at the 0.05 level, *ASI* % asymmetry index between legs, *ROM* range of motion, *DKV*: dynamic knee valgus, *CV* coefficient of variation, ES effect size, *CAI* chronic ankle instability, *PFP* patellofemoral syndrome, *ROM* range of motion.

*Indicates significant post-hoc comparison between CAI and PFP groups (p < 0.001).

^†^Indicates significant difference on post-hoc testing compared to healthy group (p < 0.001).

### Correlation

The relation between all tasks on both the involved and contralateral sides was significant (Table 6). Furthermore, an inverse moderate to good linear relation existed between ankle dorsiflexion ROM with DKV angle and LSD scores. Alternatively stated, as dorsiflexion ROM increased, the DKV angle and LSD score decreased (better movement quality). Finally, a good to excellent direct linear relation was also observed between the DKV angle and the LSD scores. That is, a greater ankle dorsiflexion ROM, DKV angle, and LSD, on the involved limb was associated with greater values on the contralateral.

**Table 6:** Spearman’s rho correlations between the different tasks.

Tasks		Ankle dorsiflexion ROM involved	DKV involved	LSD involved	Ankle dorsiflexion ROM contralateral	DKV contralateral	LSD contralateral
Ankle dorsiflexion ROM involved			− 0.667*	− 0.594*	0.737*	− 0.674*	− 0.550*
			< 0.001	< 0.001	< 0.001	< 0.001	< 0.001
DKV involved	ρ	− 0.667*		0.796*	− 0.530*	0.974*	0.707*
	*P*	< 0.001		< 0.001	< 0.001	< 0.001	< 0.001
LSD involved	ρ	− 0.594*	0.796*		− 0.494*	0.785*	0.776*
	*P*	< 0.001	< 0.001		< 0.001	< 0.001	< 0.001
Ankle dorsiflexion ROM contralateral	ρ	0.737*	− 0.530*	− 0.494*		− 0.590*	− 0.614*
	*P*	< 0.001	< 0.001	< 0.001		< 0.001	< 0.001
DKV contralateral	ρ	− 0.674*	0.974*	0.785*	− 0.590*		0.719*
	*P*	< 0.001	< 0.001	< 0.001	< 0.001		< 0.001
LSD contralateral	ρ	− 0.550*	0.707*	0.776*	− 0.614*	0.737*	
	*P*	< 0.001	< 0.001	< 0.001	< 0.001	< 0.001	

*DKV* dynamic knee valgus test, *LSD* lateral step-down test.

*Correlation is significant at the 0.01 level (2-tailed).

## Discussion

This study aimed to quantify differences in ankle dorsiflexion ROM, DKV angle, and LSD across groups as well as determine the association between limb and ankle dorsiflexion ROM, DKV angles, and the LSD ILAs scores in basketball players with CAI, PFP and healthy controls. We observed that female basketball players with PFP and CAI have impaired ankle dorsiflexion ROM, greater DKV angle, poorer scores in the LSD, and greater ILAs than healthy controls (Tables 3, 5 and Fig. 3a–c). Differences in ankle dorsiflexion ROM, DKV angle, and LSD scores in individuals with CAI and PFP were observed compared to the healthy controls, supporting our first hypothesis and substantiating the findings of previous studies^14,15,30^.

The ankle dorsiflexion ROM in the involved limb was 9° less in the CAI group and 6° less in the PFP group compared to the healthy control group (Table 3, Fig. 3a), while differences in the uninvolved limb were 4° greater in the CAI compared to the PFP group and 7°less in the PFP compared to healthy controls. These ROM findings agree with Grindstaff et al.^14^, who reported that patients with CAI had reduced ankle dorsiflexion ROM compared to healthy subjects^14^. Similarly, in the study by Rabin et al.^15^ movement pattern was assessed visually during an LSD and rated as "good" or "moderate in PFP patients^15^. They demonstrated that ankle dorsiflexion ROM was more limited among participants with a moderate versus a good quality of movement^15^. Importantly, our ankle dorsiflexion ROM values exceeded the previously established minimum detectable difference of 4° and 6°, suggesting these differences may impact patient function^14,41^.

The DKV angle in the single-leg squat test revealed that the involved limb angle was nearly 8° greater in the CAI group and approximately 9° greater in the PFP group compared to healthy controls (Table 3, Fig. 3b). The DKV angle for the contralateral limb was almost 5° greater in the CAI and 6° greater in the PFP versus the healthy control group (Table 3, Fig. 3b). The minimal detectable change (MDC) of the DKV angle ranged from approximately 3.5–10° for the single-leg squat^48^. Accordingly, the mean difference observed in patients with CAI and PFP of over 8° between groups is clinically relevant and in agreement with the results of previous investigators who demonstrated greater DKV angle in females with than without PFP^49^. Although many studies emphasize that DKV angle is a significant risk factor in patients with PFP^7^, to our best knowledge, only one study^8^ has confirmed that patients with CAI demonstrated increased DKV angle compared with coper and control subjects^8^. The paucity of studies investigating DKV angle in CAI offers future research opportunities. Additionally, these results collectively suggest the need to evaluate and improve ankle dorsiflexion ROM as well as DKV angle in patients with both PFP and CAI.

In terms of LSD results, our study revealed that CAI and PFP performed, on average, moderate quality movement (3.45), while healthy groups performed on average good quality movement (0.55) (Table 3, Fig. 3c). On the contralateral side, we observed moderate quality movement for CAI and PFP groups (1.40, 2.10 respectively) and good quality movement for the healthy group (0.25). There is a paucity of studies directly comparing the scores of movement quality between asymptomatic and musculoskeletal pain individuals. Ferreira et al. showed that women with PFP show muscle coordination and motor control alterations, which correlate with kinematic alterations during LSD^50^. There is, however, limited research investigating LSD in women with CAI. In one study that was conducted, the authors reported individuals with poor movement quality had significantly less ankle dorsiflexion ROM compared to the good movement quality group^14^.

Our second hypothesis was only partially supported as contralateral ankle dorsiflexion ROM was 4° greater in the CAI (44°) compared to the PFP group (40°) (Table 3, Fig. 3a). In rehabilitation, inter-clinician changes of 4.6° and intra-clinician changes of 4.7° are needed for a change in dorsiflexion ROM to be considered outside of the measurement error of the WBLT^51^. Our data did not exceed the MDC; accordingly, the current study’s findings should be treated with caution^51^. Moreover, in our study, the difference in ankle dorsiflexion ROM did not affect the significance of the difference between the DKV angle and LSD scores in both groups observed on the contralateral side, contrasting other previous evidence^14,40^. For example, Grindstaff et al.^14^ showed that patients with CAI presenting a poor movement quality had, on average, 6° less ankle dorsiflexion ROM than participants with good movement quality and almost 3° less than the participants with moderate movement quality in involved and contralateral side. Also, Rabin et al. showed that decreased ankle dorsiflexion ROM impacted the movement quality of the LSD in healthy females^40^. A possible explanation of the study discrepancy could be related to the inter-individual variability of neuromuscular control observed among patients with musculoskeletal pain^52^. Furthermore, the lack of studies directly comparing patients with PFP and CAI makes comparing our results and other evidence difficult. Thus, future studies should investigate the combined role of kinematic, muscle activation, strength, and movement quality deficits in individuals with CAI and PFP to determine whether dorsiflexion ROM and kinematic asymmetry exist in these populations impacting functional performance.

Our asymmetry analyses revealed a significant association between ankle dorsiflexion ROM and DKV with the LSD scores in female basketball athletes with PFP and CAI in support of our third hypothesis (Tables 3, 5). Our research agrees with Herrington, who demonstrated that healthy national league basketball players showed significant asymmetry in DKV angle during bilateral drop jump landings^53^. Moreover, a study examining the performance of patients with PFP during the single-leg squat and Y-balance test showed results consistent with our findings^30^. They showed that male military recruits with greater asymmetry on the Y-Balance test posterolateral direction, and frontal plane knee projection angle were at a greater risk of developing PFP^30^. This asymmetry may suggest a neuromuscular imbalance between limbs. Side-to-side imbalances in neuromuscular strength, flexibility, and coordination represent significant predictors of increased injury risk^54^. Asymmetry between two limbs is considered a risk factor in athletes^28^. In the present study, comparing athletes with PFP and CAI, it seems that athletes with PFP experience more asymmetry and motor deficits on the contralateral side. Therefore, including the entire motor chain on both lower limbs is significant in evaluating and designing prevention and treatment programs. In addition, further prospective studies are needed to explore whether there is a cause-effect relationship between inter-limb asymmetry and injury development.

In addition, we aimed to determine which task has the greatest sensitivity to identify asymmetries based on ASI calculation. ASI varied among ankle dorsiflexion ROM, DKV angle, and LSD tasks and indicated that the percentage of asymmetry could differ depending on the task performed. Accordingly, this finding indicates that obtaining measurements on various independent tasks is important to assess ASI. The largest ASI detected in our study was found in DKV for CAI and PFP. This ASI is higher than the 10–15% threshold describing the potential risk of injury among participants as reported in the literature^28^. Therefore, the DKV is likely a critical task for detecting ILA between legs. In addition, prospective research is needed to determine if there is a direct correlation between DKV angle asymmetry and injury rate over time. Moreover, it is challenging to establish an ASI threshold for typical values on the other tasks examined in this research because of the scarcity of research on this topic.

The fourth hypothesis’s results demonstrated a moderate to good relationship between ankle dorsiflexion ROM and LSD scores and a good to strong relationship between DKV angle and LSD scores. These findings agree with similar studies investigating ankle dorsiflexion ROM and LSD scores in CAI and PFP^14,15,21,40^. During the LSD, maximal ankle dorsiflexion ROM is required to allow the heel to remain in contact with the ground^14^. A limitation in ankle dorsiflexion ROM or the proximal lower limb joints may require compensation from other joints^16^. Decreased ankle dorsiflexion ROM will limit the forward progression of the tibia over the talus, resulting in compensatory subtalar pronation, which, in turn, may displace the knee medially^55^. Previous studies suggest that limited ankle dorsiflexion ROM may be associated with a greater medial knee displacement during various functional activities^16–18^; such compensation may increase DKV angle. Less research has been conducted to investigate the influence of limited ankle dorsiflexion ROM on a frontal and transverse plane lower limb alignment. Bell et al. found that ankle ROM and strength rather than hip ROM and strength were associated with DKV angle during a controlled squat^56^. In the above study, the DKV angle was corrected by adding a heel lift, confirming the role of limited ankle dorsiflexion ROM in dynamic knee valgus during a loading task^56^. Bell-Jenje et al.^21^ demonstrated that participants with restricted dorsiflexion ROM had greater dynamic knee valgus during the LSD^21^. Also, they showed that the association between an increase in hip adduction ROM and decreased dorsiflexion ROM during a functional loading task such as the LSD test emphasizes the importance of the kinetic chain in lower limb alignment^21^. In the closed kinetic chain, the relative angle, magnitude, and direction of one motion-dependent body segment affect another segment, leading to differences in how these segments interact^14,21^. This finding is significant in a clinical context where limited dorsiflexion ROM is associated with CAI^14^, PFP^15^, and dorsiflexion ROM may, therefore, play a key role in predicting injuries^21^, but further longitudinal research is needed. Additionally, these results collectively suggest the need to evaluate and improve ankle dorsiflexion ROM to improve movement quality in patients with both PFP and CAI. There are few studies investigating this relationship in CAI. So, future research is needed to investigate a relationship between CAI and other lower limb injuries during functional tasks (e.g. jump landing/cutting test, single leg countermovement jumps); possible use of posture software for the movement analysis is further suggested. Also, a future prospective study is needed to examine whether increased DKV angle with reduced dorsiflexion ROM may contribute to a greater risk of knee injury in patients with CAI.

### Limitations

This study presents some limitations that should be acknowledged. Firstly, we cannot obtain causative conclusions from our study by adopting a cross-sectional design^57^. Accordingly, prospective studies should consider the cause-effect relationship between inter-limb asymmetry and injury development. Secondly, we have recruited only a sample of female non-professional basketball players with CAI and PFP. Thus, our findings could not be generalized to male participants of different ages, professional athletes, players of other contact sports or in the presence of different clinical conditions (e.g. ACL reconstruction). Thirdly, the small effect size and power used in sample size estimation is another limitation that should be considered in future research. Fourthly, this study did not examine hormone profiles and status or inquire about contraception usage, which can have important implications for physiological function in female participants^58^. Lastly, we assessed the quality of movement only using the LSD and adopted a 2D analysis of limb motion. Therefore, future studies should consider investigating a motor task similar to basketball-specific movement techniques (e.g. jump landing/cutting test, single-leg countermovement jumps) and also integrating a 3D analysis (e.g. hip, knee and ankle joints simultaneously) combined with a real-time electromyography data acquisition to gain further insight into the importance of ankle dorsiflexion ROM and DKV angle during sport-specific tasks.

## Conclusions

Female basketball players with PFP and CAI have impaired ankle dorsiflexion ROM, greater DKV angles, poorer scores in the LSD, and greater ILAs compared to healthy controls. Ankle dorsiflexion ROM and DKV were associated with LSD. These findings inform clinicians and researchers of the need to evaluate ROM and function throughout the kinetic chain and in both lower limbs in injured athletes tailoring specific rehabilitation programs.

## Data Availability

The datasets generated during and/or analyzed during the current study are available from the corresponding author on reasonable request.
